# Pseudorabies Virus Infection Causes Downregulation of Ligands for the Activating NK Cell Receptor NKG2D

**DOI:** 10.3390/v13020266

**Published:** 2021-02-09

**Authors:** Sofie Denaeghel, Steffi De Pelsmaeker, Cliff Van Waesberghe, Herman W. Favoreel

**Affiliations:** Laboratory of Immunology, Department of Virology, Parasitology and Immunology, Faculty of Veterinary Medicine, Ghent University, 9820 Merelbeke, Belgium; sofie.denaeghel@ugent.be (S.D.); steffi.depelsmaeker@gmail.com (S.D.P.); cliff.vanwaesberghe@ugent.be (C.V.W.)

**Keywords:** pseudorabies virus, NKG2D, ligands, pULBP1, pMIC2, pig

## Abstract

Herpesviruses display a complex and carefully balanced interaction with important players in the antiviral immune response of immunocompetent natural hosts, including natural killer (NK) cells. With regard to NK cells, this delicate balance is illustrated on the one hand by severe herpesvirus disease reported in individuals with NK cell deficiencies and on the other hand by several NK cell evasion strategies described for herpesviruses. In the current study, we report that porcine cells infected with the porcine alphaherpesvirus pseudorabies virus (PRV) display a rapid and progressive downregulation of ligands for the major activating NK cell receptor NKG2D. This downregulation consists both of a downregulation of NKG2D ligands that are already expressed on the cell surface of an infected cell and an inhibition of cell surface expression of newly expressed NKG2D ligands. Flow cytometry and RT-qPCR assays showed that PRV infection results in downregulation of the porcine NKG2D ligand pULBP1 from the cell surface and a very substantial suppression of mRNA expression of pULBP1 and of another potential NKG2D ligand, pMIC2. Furthermore, PRV-induced NKG2D ligand downregulation was found to be independent of late viral gene expression. In conclusion, we report that PRV infection of host cells results in a very pronounced downregulation of ligands for the activating NK cell receptor NKG2D, representing an additional NK evasion strategy of PRV.

## 1. Introduction

Members of the natural killer group 2 (NKG2) receptor family all are type II transmembrane glycoproteins, belonging to the C-type lectin-like family [1]. The activating receptor NKG2D (CD314) is constitutively expressed in natural killer (NK) cells of different species—including humans, mice and pigs—and associates with itself to form a disulfide-linked homodimer [2]. When triggered, signals of the NKG2D receptor are transmitted through association with adaptor molecules like DAP10 and/or DAP12 [3,4,5]. Expression of NKG2D, DAP10 and DAP12 has been described also in porcine NK cells [6,7,8].

Ligands for NKG2D are stress-induced, and upregulation of these ligands can be observed in damaged, transformed and/or virus-infected cells [9]. NKG2D ligand expression is initiated by various pathways, with the DNA damage response pathway being the most prominent [10,11]. All described NKG2D ligands show homology to major histocompatibility complex (MHC) class I molecules and belong to either of two groups: MHC I chain-related proteins (MIC) on the one hand and retinoic acid transcript 1/UL16 binding proteins (REAT1/ULBP1) on the other hand. Ligands for human NKG2D include MICA, MICB and ULPB1-6. In mice, three ligand families for murine NKG2D have been identified: RAE-1, minor histocompatibility protein 60 (H60) and mouse UL-16 binding protein-like transcript 1 (MULT-1) [12,13]. Human ULBP1-3 and -6, as well as mouse RAE1α-ε and H60c, are GPI-anchored proteins, whereas the other ligands are transmembrane proteins [10]. In pigs, only two ligands have been described thus far, porcine MIC2 (pMIC2) and porcine ULPB1 (pULBP1). The latter shows a 35%–54% amino acid sequence homology to human ULPBs, whereas the former shows an amino acid sequence homology of 54% to human MICA [14,15,16,17]. Both molecules have been reported to have the ability to bind (human) NKG2D, and it appears likely that there are additional, yet unidentified porcine ligands for NKG2D [17].

The balance in activating/inhibitory signals received by an NK cell determines its activity. As NKG2D binding favors activation, modulation of NKG2D ligand expression may serve as an NK cell evasion mechanism. Herpesviruses of all three subfamilies have developed different evasion strategies to suppress NKG2D-mediated recognition of infected cells [18,19,20]. The porcine pseudorabies virus (PRV) belongs to the alphaherpesviruses, the largest subfamily of herpesviruses, which also includes three human herpes viruses: herpes simplex virus types 1 and 2 (HSV1 and HSV2) and varicella-zoster virus (VZV) [21]. NKG2D ligand expression has been investigated on VZV- and HSV1-infected cells [22,23]. Infection of cells with HSV1 leads to reduced expression of NKG2D ligands MICA, ULBP1, ULPB2 and ULBP3 on the cell surface. It is not clear if this cell surface downregulation corresponds with an alteration in protein level, since independent studies show different results. Flow cytometric analysis of the level of MICA in infected HeLa cells showed an unchanged total protein level [22], although Western blot assays on infected ARPE-19 and human foreskin fibroblast cells suggested the opposite, indicating a reduction of the total MICA cellular protein level [23]. The latter study reported reduced protein levels for ULBP2 and ULBP3, but not ULBP1. As indicated by the authors, the usage of different analysis methods and different cell types could contribute as possible causes of these contradictory results [22,23]. The mechanisms that lead to suppression of NKG2D ligand expression in HSV1-infected cells are still unclear, although late viral gene products seem to be involved in downregulation of MICA from the cell surface [22]. There is also evidence that miR-H8, an HSV1-encoded miRNA, targets the GPI anchored pathway, thereby significantly reducing expression levels of ULBP2 and ULBP3, but not MICA/B and ULBP1 [24]. For VZV, the picture is less clear, as VZV infection of cells was found to lead to downregulation of ULBP2 and ULBP3, but upregulation of MICA [23]. Although downregulation of NKG2D ligands as described in HSV1-infected cells is likely to result in reduced binding of NKG2D to the surface of infected cells, to the best of our knowledge, no reports have assessed the binding efficiency of NKG2D to alphaherpesvirus-infected cells.

We reported earlier that the porcine alphaherpesvirus PRV suppresses NK cell activity via gD-mediated downregulation of ligands for the activating NK cell receptor DNAM-1 and US3-mediated upregulation of ligands for the inhibitory NK cell receptor CD300a [25,26], but it is currently unknown if PRV infection results in altered expression of NKG2D ligands. Therefore, in the current study, we investigated the effect of PRV infection on NKG2D binding to the surface of infected cells and on the expression of NKG2D ligands.

## 2. Materials and Methods

### 2.1. Infection of Swine Kidney (SK) Cells

**Figure 1:**
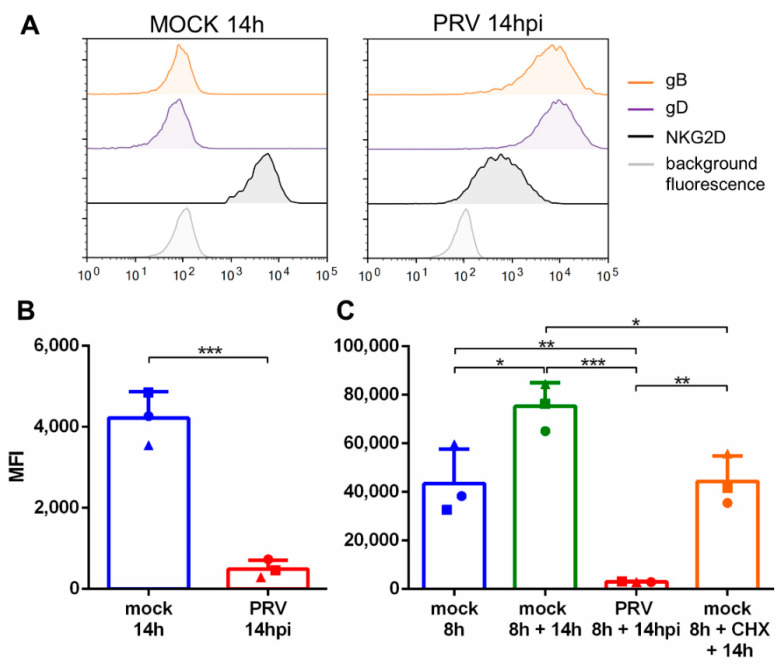
Pseudorabies virus (PRV) infection suppresses NKG2D ligand expression on the surface of infected cells. (**A**) Flow cytometric histograms of mock-infected (left) and PRV-infected (right) swine kidney (SK) cells at 14 hpi, stained with antibodies against viral proteins gB (orange) and gD (purple), incubated with recombinant Fc-tagged human NKG2D protein (black) or a secondary antibody control (gray). (**B**) SK cells in suspension were mock-infected or infected with PRV strain Kaplan and at 14 h post-inoculation (hpi) incubated with recombinant Fc-tagged human NKG2D protein and analyzed by flow cytometry. (**C**) SK cells were cultivated in suspension for 8 h before mock or PRV inoculation. If indicated, cycloheximide (CHX) was added at 8 h of cultivation at a concentration of 5 µg/mL. At the different time points, indicated in the graph, cells were incubated with recombinant Fc-tagged human NKG2D protein and analyzed using flow cytometry. Graphs show the median fluorescence intensity (MFI) for each condition. Bars represent the mean value ± SD; different symbols correspond to individual data points from different independent experiments, *n* = 3. Statistically significant differences are indicated with asterisks (* *p* < 0.05 ** *p* < 0.01, *** *p* < 0.001).

SK cells were cultured in modified Eagle’s medium (MEM), supplemented with 10% (*v*/*v*) inactivated fetal bovine serum (FBS), 100 U/mL penicillin, 100 µg/mL streptomycin and 0.05 mg/mL gentamycin (all from Gibco, Thermo Fisher Scientific, Waltham, MA, USA). SK cells grown in monolayers were detached from cell culture flasks (175 cm^2^) using trypsin, seeded in suspension culture flasks (25 cm^2^, Sarstedt, Nümbrecht, Germany) at 1.2 × 10^7^ cells/8.5 mL, and put on a rocking platform at 37 °C as described before [25]. SK cells were either directly or after 8 h inoculated with PRV strain Kaplan [27] at a multiplicity of infection (MOI) of 10 and put back on the rocking platform at 37 °C for 14 h. To ensure that this inoculation procedure led to a 100% infection efficiency, flow cytometric detection of viral glycoproteins gB and gD was performed in every experiment (illustrated in Figure 1A). For the experiment using phosphonoacetic acid (PAA, Sigma-Aldrich, St. Louis, MO, USA, catalog number 284270), SK cells were treated with PAA at a concentration of 400 µg/mL from 30 min prior to infection onwards as described before [28]. For the experiment using cycloheximide (CHX, Sigma-Aldrich, catalog number C1988), SK cells were treated with CHX at a concentration of 5 µg/mL at 8 h of cultivation.

### 2.2. Analysis of Cell Surface Binding of NKG2D and Cell Surface Expression of NKG2D Ligands via Flow Cytometry

For flow cytometry analysis, cells were washed in PBS. All incubation steps were performed in 96-well V-bottomed plates for 40 min at 4 °C in PBS. Cells were washed two times in PBS between each step. Prior to the pULBP1 staining, cells were fixed in 2% paraformaldehyde for 10 min at room temperature. The different combinations of primary antibodies and secondary reagents used for each assay are listed in Table 1 and antibodies were diluted in PBS. The binding assay of recombinant human NKG2D to porcine cells has been described before [17]. Control stainings were typically performed using only secondary antibody. An additional control for NKG2D binding assays consisted of a binding assay using a human IgG1 Fc control protein (Adipogen, Liestal, Switserland, catalog number AG-35B-0007-C050). For the pULBP1 staining, a mouse IgM was used as an isotype control (Invitrogen, Thermo Fisher Scientific, Waltham, MA, USA catalog number 14-4752-82). Viability of the cells was assessed by propidium iodide staining (Invitrogen, catalog number P3566) or Sytox Blue staining (Invitrogen, catalog number S34857). Live cells were gated and used for further analyses. Series of flow cytometry experiments were performed using a FACS Aria III (BD-Biosciences, San Jose, CA, USA) or a NovoCyte Flow Cytometer (ACEA Biosciences, Agilent, Santa Carla, CA, USA), depending on the availability of the devices, explaining the differences in arbitrary units of fluorescence intensity and the use of different live/dead cell stains. Samples were analyzed with NovoExpress software (ACEA Biosciences). Median fluorescence intensity was determined by subtracting the median fluorescence intensity of the control staining from the median fluorescence intensity of each sample.

### 2.3. Real Time-Quantitative PCR (RT-qPCR)

RNA isolations were done as described before [28]. An RNeasy minikit (Qiagen, Hilden, Germany, catalog number 74104) was used according to the manufacturer’s protocol to isolate RNA. Purified RNA was treated with DNase I (RNase free, New England Biolabs, catalog number M0303S) at 37 °C for 10 min. To stop the DNase I activity, EDTA (Invitrogen, final concentration of 5 mM) was added, followed by an incubation of the RNA at 75 °C for 10 min. Quality and quantity of the RNA yields were analysed using a DeNovix DS-11 FX spectrophotometer. To obtain complementary DNA (cDNA), an iScript cDNA synthesis kit (Bio-Rad, Hercules, CA, USA, catalog number 1708891) was used according to the manufacturer’s instructions. Five hundred nanograms of DNA-free total RNA was used for the reverse transcription (RT). The cycle conditions were 1 cycle of 5 min at 25 °C (priming), 20 min at 46 °C (reverse transcription), and 1 min at 95 °C (RT inhibition). Primer oligonucleotides were synthesized by Integrated DNA Technologies (IDT). The sequences of the forward and reverse primers used for the amplification of the genes of interest can be found in Table 2. For non-pre-existing primer oligonucleotide sequences, the primer design tool Primer-BLAST (NIH, Brethesda, MD, USA) was used. For quantitative PCRs (qPCRs), the PCR reaction mix consisted of the SYBR green PCR master mix (Applied Biosystems, Thermo Fisher Scientific, Waltham, MA, USA, catalog number 4309155), 0.5 µM of the forward and reverse primer and cDNA. In a final volume of 20 µL qPCRs were performed on MicroAmp Fast optical 96-well reaction plates (Applied Biosystems, Thermo Fisher Scientific, catalog number 4346906) using a StepOnePlus real-time PCR system (Applied Biosystems, Thermo Fisher Scientific, catalog number 4376600). The relative expression of the target genes (pULBP1 and pMIC2) was analyzed using the double delta threshold cycle method and normalized to the level of expression of the 28S rRNA gene, which has been validated and used as a reference gene in other similar gene expression analyses on PRV-infected cells [28,30].

### 2.4. Statistical Analysis

Statistical analysis was performed using Graphpad Prism 6.0 (San Diego, CA, USA). Data were analyzed for statistical differences with a one-way analysis of variance (ANOVA) or unpaired *t*-test at the 5% significance level. Post hoc comparisons between different conditions were performed using Tukey’s range test.

## 3. Results

### 3.1. PRV Infection Suppresses NKG2D Ligand Expression on the Surface of Infected Cells

To determine if PRV infection in swine kidney (SK) cells affects the binding of NKG2D to (and thus the expression of NKG2D ligands on) the cell surface, we investigated the binding of recombinant NKG2D to the surface of mock- or PRV-infected SK cells. Binding assays were performed at 14 hpi by means of flow cytometry. In line with what is known for human NKG2D ligands like MICA [31], we found that NKG2D ligands on the cell surface of porcine SK cells are trypsin- and accutase-sensitive and therefore cannot be detected immediately after detachment of adherent cells via trypsinization (data not shown). However, we found that NKG2D ligands are rapidly re-expressed upon SK cell cultivation in suspension via procedures described earlier [25,26,32,33,34], resulting in robust NKG2D binding to SK cells cultivated in suspension for, e.g., 14 h (Figure 1A,B). Hence, to analyze NKG2D binding to the cell surface of mock-and PRV-infected cells, SK cells grown in monolayers were detached using trypsin, cultivated in suspension and either inoculated with PRV at an MOI of 10 or not inoculated. In line with earlier reports [25,26,32,33,34], this inoculation procedure led to 100% infection efficiency, as determined by flow cytometric analysis of SK cells stained at 14 hpi for PRV glycoproteins gB and gD (Figure 1A). Interestingly, PRV-infected cells showed a virtual lack of NKG2D binding at 14 hpi, whereas mock-infected cells analyzed at the same time point showed robust NKG2D binding (Figure 1A,B). Since mock-infected cells showed a virtual absence of NKG2D binding when analyzed directly upon trypsinization, this indicates that the cultivation of SK cells in suspension triggered substantial expression of NKG2D ligands, which was abolished in PRV-infected cells. Hence, the results of these assays suggest that PRV infection prevents expression of new NKG2D ligands on the cell surface of infected SK cells. To investigate if PRV infection also results in downregulation of pre-existing NKG2D ligands on the cell surface, the experimental set-up was slightly altered. In preliminary experiments, we had noticed that NKG2D ligand expression in SK cells cultivated in suspension gradually increases until at least 24 h of cultivation (data not shown). Therefore, we decided to inoculate SK cells at an intermediate time point, 8 h of cultivation in suspension, when SK cells show clear but not yet maximal expression of NKG2D ligands, and analyzed cells again at 14 hpi. Figure 1C shows that in mock-infected SK cells, NKG2D ligand expression indeed increases over time, since NKG2D binding is significantly increased in cells cultivated for 22h (8 h + 14 h) compared to cells cultivated for 8 h. Importantly, in cells inoculated with PRV at 8 h of cultivation and analyzed at 14 hpi, NKG2D binding was not increased but was even significantly decreased compared to cells cultivated for 8 h. This assay suggests that PRV infection not only prevents the de novo expression of NKG2D ligands on the cell surface but also triggers downregulation of NKG2D ligands that are already present on the cell surface. As an additional control, an Fc (human) IgG1 control protein was used, showing similar results to when using only secondary antibody (Supplementary Figure S1).

To confirm that the significantly lower NKG2D binding to SK cells cultivated for 8 h followed by PRV infection for 14 h compared to binding to SK cells cultivated for 8 h could not merely be explained by gradual turnover of NKG2D cell surface ligands from 8 h to 22 h of cultivation, an additional experimental condition was tested. Here, at 8 h of cultivation in suspension, SK cells were treated with cycloheximide (CHX, used at a concentration of 5 µg/mL as described before [35]) to prevent the production of new NKG2D ligands, allowing us to assess whether gradual turnover of NKG2D ligands may lead to reduced binding of NKG2D to cells 14 h later. As shown in Figure 1C, although cultivating SK cells for another 14 h in suspension in the presence of CHX prevented the increase in NKG2D binding observed in the absence of CHX, as expected, it did not significantly reduce NKG2D binding. Hence, we can conclude that PRV infection indeed not only prevents the de novo expression of NKG2D ligands on the cell surface, but also triggers downregulation of NKG2D ligands that are already present on the cell surface of infected cells. To investigate if (viral tegument proteins of) incoming PRV virions contribute to the downregulation of NKG2D ligands, UV-inactivated virus was used to inoculate SK cells and NKG2D binding was checked as mentioned before. We found that UV-inactivated virus was unable to trigger the downregulation of NKG2D ligands (Supplementary Figure S2).

### 3.2. PRV Infection Leads to a Fast, Gradual and Persistent Downregulation of NKG2D Ligands

**Figure 2:**
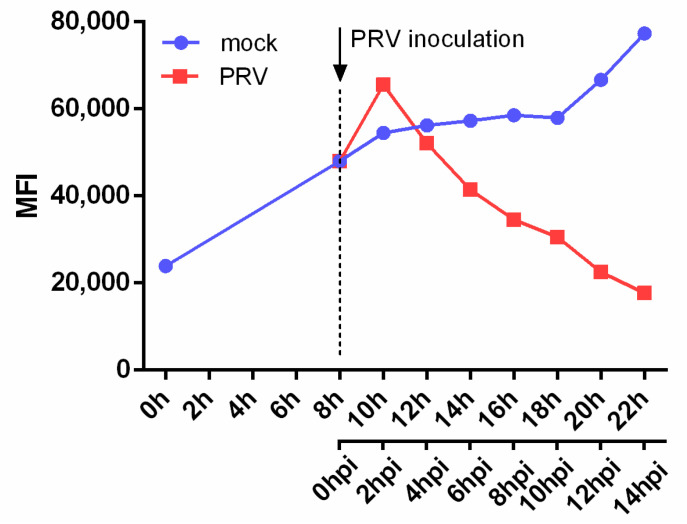
Time course of the effect of PRV or mock inoculation on NKG2D binding to SK cells. SK cells were cultivated in suspension for 8 h before mock or PRV inoculation. At different time points, as shown in the graph, SK cells were incubated with recombinant Fc-tagged human NKG2D protein and analyzed using flow cytometry. The graph shows the median fluorescence intensity (MFI) for each condition. Representative graph of 2 independent experiments is shown, *n* = 2.

To investigate when the observed downregulation of NKG2D ligands occurs during PRV infection, a time course assay was performed. Again, SK cells were cultivated in suspension and at 8 h of cultivation, either inoculated with PRV or not inoculated, followed by flow cytometric analysis of NKG2D binding every 2 h. Figure 2 shows a steady rise in NKG2D ligand expression on the surface of mock-infected SK cells. In PRV-infected cells on the other hand, a rapid but very brief increase in NKG2D binding can be observed at 2 hpi, which is followed by a steep and progressive decrease in NKG2D binding at all subsequent time points. Hence, whereas PRV infection initially triggers a (likely stress-induced) increase in NKG2D ligand expression, this increase is very brief and rapidly followed by an efficient and progressive decrease in NKG2D ligand expression on the cell surface of infected cells.

### 3.3. Downregulation of NKG2D Ligands Is Independent of Late Viral Gene Expression

**Figure 3:**
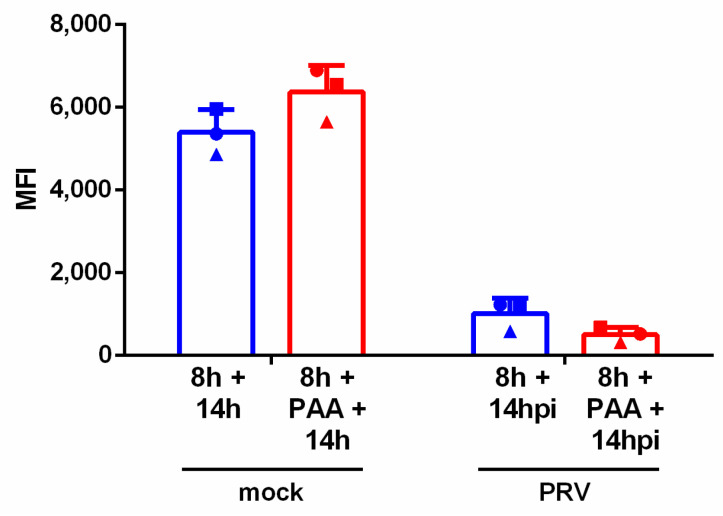
Viral late gene expression is not required for PRV-induced downregulation of NKG2D ligand expression. SK cells were cultivated in suspension for 8 h before mock or PRV infection. SK cells were treated or untreated with phosphonoacetic acid (PAA, 400 µg/mL) starting 30 min before inoculation. All samples were incubated with recombinant Fc-tagged human NKG2D protein at 14 hpi and analyzed using flow cytometry. Graph shows the median fluorescence intensity (MFI) for each condition. Bars represent the mean value ± SD; different symbols correspond to individual data points from different independent experiments, *n* = 3.

To investigate whether the expression of late viral genes is involved in the observed downregulation of NKG2D ligands, the DNA polymerase inhibitor phosphonoacetic acid (PAA) was used, as described before [36]. This inhibitor prevents the expression of late genes, since expression of these depends highly on viral genome replication. Like before, SK cells were cultivated in suspension for 8 h before PRV inoculation. PAA treatment (400 µg/mL) was initiated from 30 min before mock or PRV inoculation up to analysis at 14 hpi. Untreated cells served as a control. Successful PAA treatment was confirmed by Western blot, as assessed by expression of US3 (early protein) and lack of expression of gE (late protein) as described earlier [28,35,37] (data not shown). Figure 3 shows that addition of PAA did not affect PRV-induced downregulation of NKG2D ligand expression. Hence, expression of late genes is not required for downregulation of NKG2D ligands in PRV-infected SK cells.

### 3.4. pULBP1 Expression Is Reduced after PRV Infection

**Figure 4:**
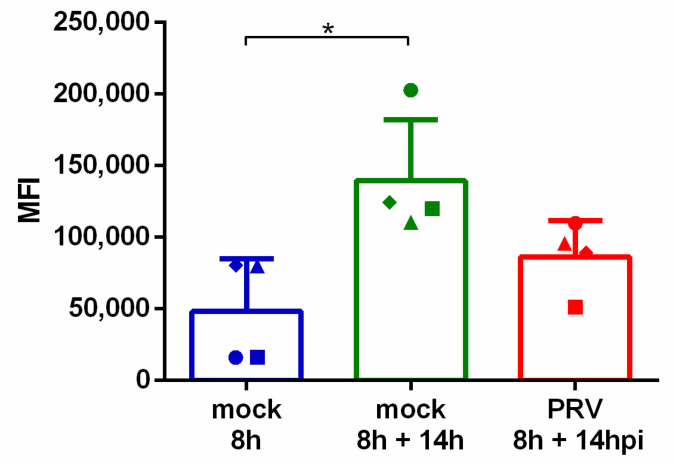
pULBP1 expression on the cell surface is reduced after PRV infection. SK cells were cultivated in suspension for 8 h before mock or PRV inoculation. At the indicated time points, pULBP1 expression on the cell surface was measured using flow cytometry. Graphs show the median fluorescence intensity (MFI) for each condition. Bars represent the mean value ± SD; different symbols correspond to individual data points from different independent repeats, *n* = 4. Statistically significant differences are indicated with asterisks (* *p* < 0.05).

The impact of PRV infection on the expression of the major confirmed porcine NKG2D ligand, pULBP1 [16], on the cell surface of SK cells was examined. To this end, SK cells were cultivated in suspension for 8 h, subsequently mock- or PRV-infected, stained for pULBP1 at 14 hpi and analyzed using flow cytometry. Cultivation of SK cells in suspension for 22 h compared to 8 h resulted in an increase in pULBP1 cell surface expression (Figure 4). PRV infection resulted in a noticeable and reproducible (yet not statistically significant) downregulation of this NKG2D ligand in comparison with mock-infected cells at the corresponding time point (22 h of cultivation).

### 3.5. PRV Infection Results in a Fast and Progressive Downregulation of pULBP1 and pMIC2 mRNA Expression

**Figure 5:**
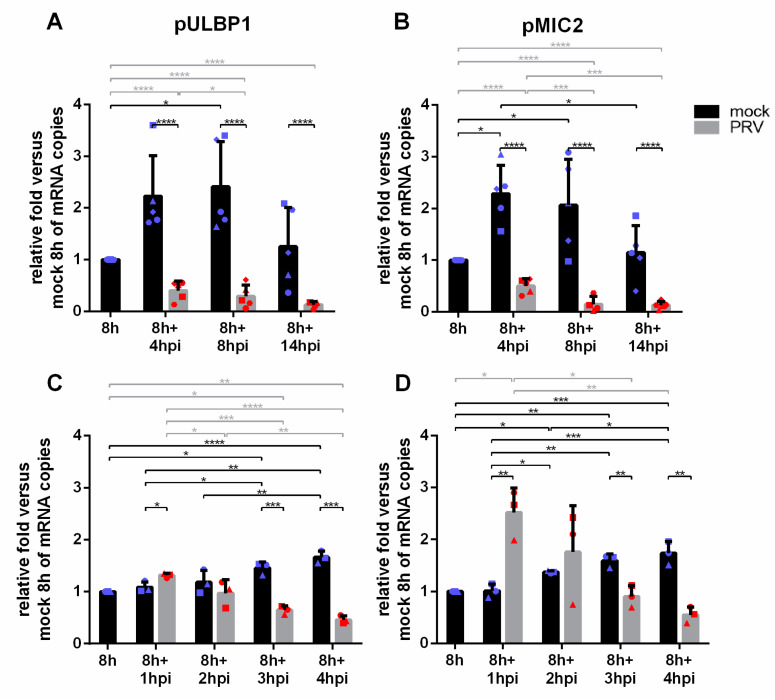
mRNA expression of pULBP1 and pMIC2 in SK cells upon mock or PRV infection. Gene expression of (**A**) pULBP1 and (**B**) pMIC2 were analyzed in mock-infected SK cells or PRV-Kaplan-infected SK cells at different time points as shown in the graph, and were normalized to gene expression in SK cells cultivated in suspension for 8 h. Graphs represent the mean and SD values of relative mRNA expression obtained from five (**A**,**B**) or three (**C**,**D**) independent repeats of the experiment, *n* = 5 or *n* = 3. Statistically significant differences are indicated with asterisks (* *p* < 0.05, ** p < 0.01, *** *p* < 0.001, **** *p* < 0.0001).

Although pULBP1 is the major confirmed porcine ligand of NKG2D, pMIC2 has also been suggested to serve as a potentially minor porcine NKG2D ligand [16,17]. Since an antibody to detect pMIC2 protein levels is not available, we evaluated the effect of PRV infection on mRNA expression of pULBP1 and pMIC2 via reverse (RT)-quantitative PCR (qPCR). Again, SK cells cultivated for 8 h in suspension were mock- or PRV-infected. At different time points post-inoculation (4 hpi, 8 hpi and 14 hpi), gene expression was analyzed in mock-infected and PRV-infected SK cells (Figure 5A,B). Results were normalized to expression of the genes in SK cells at 8 h of cultivation in suspension, the time point at which cells were mock-inoculated or inoculated with PRV. In mock-infected cells, an increase in the expression of both genes was observed from the original time point (8 h of cultivation in suspension) onwards, except for the last time point tested (8 h + 14 hpi cultivation). More importantly, PRV infection resulted in a progressive downregulation of mRNA of both genes, starting from the earliest time point (4 hpi). Since the kinetic time course of NKG2D binding (Figure 2) showed a rapid spike of NKG2D binding at 2 hpi, followed by a rapid and progressive decrease in NKG2D binding at later time points, we repeated the RT-qPCR assays for pMIC2 and pULBP1 at earlier time points post-inoculation (1 hpi, 2 hpi, 3 hpi, 4 hpi). Interestingly, Figure 5C,D show that the expression of pMIC2 showed a rapid increase (1 hpi), followed by a gradual and progressive decrease, largely in line with the NKG2D binding assays, whereas pULBP1 showed a gradual and progressive decrease at all time points.

## 4. Discussion

In the current study, we report (i) that infection of SK cells with the porcine alphaherpesvirus pseudorabies virus (PRV) results in a downregulation of NKG2D ligand surface expression; (ii) that PRV-induced downmodulation of NKG2D ligands is fast and persistent; (iii) that this NKG2D ligand downregulation is independent of late viral gene expression; (iv) that PRV infection appears to result in downregulation of pULBP1, the major confirmed porcine NKG2D ligand, from the cell surface; and (v) that PRV infection results in downregulation of gene expression of pULBP1 and pMIC2, another potential NKG2D ligand.

Although a wide range of NK cell evasion mechanisms have been described for betaherpesviruses over the past decades [20], relatively few alphaherpesviruses’ NK cell evasion mechanisms have been described [20,22,23,25,26,38,39]. For PRV, expression of the gD protein, either in PRV-infected cells or gD-transfected cells, results in downregulation and degradation of CD122, a ligand for the activating NK cell receptor DNAM-1 [25]. In addition, expression of the PRV US3 kinase in infected cells has been reported to trigger increased binding of the inhibitory NK cell receptor CD300a to the cell surface of infected cells [26]. In the current study, we report a third PRV NK evasion mechanism, consisting of NKG2D ligand downregulation. Other alphaherpesviruses like HSV1 and VZV have also been reported to affect the expression of NKG2D ligands on the surface of infected cells [22,23]. HSV1 infection, just like PRV infection, results in downregulation of NKG2D ligands, whereas VZV infection triggers both up- and downregulation of certain NKG2D ligands. Studies investigating these modifications of NKG2D ligand expression are based on flowcytometric antibody detection of specific NKG2D ligands [22,23]. NKG2D binding assays, like those performed in the current study, could be useful to confirm whether other alphaherpesviruses, like PRV, trigger decreased NKG2D ligand binding to infected cells.

Overall, the mechanisms underlying alphaherpesvirus-induced NKG2D ligand downregulation remain incompletely understood. Much more information on NKG2D evasion in herpesviruses can be found in the subfamily of the betaherpesviruses [20]. An array of different mechanisms are used by these viruses to establish NKG2D ligand downregulation, relying on different viral proteins and miRNA strategies. Retention of newly synthesized NKG2D ligands in the endoplasmatic reticulum (ER)/Golgi apparatus has been described for cytomegaloviruses in humans [40,41,42,43], mice [44] and rhesus macaques [45]. Targeting NKG2D ligands for lysosomal or proteosomal degradation constitutes another mechanism to downregulate NKG2D ligands and has been described for human cytomegalovirus (HCMV) [46,47] and human herpes virus 6 (HHV6) [48]. HCMV has also been reported to trigger NKG2D ligand downregulation via microRNAs (miRNAs) like miR-UL112 [49,50,51].

NKG2D ligand downmodulation during PRV infection was not affected by the presence of PAA, indicating that late viral gene expression is not involved. This contrasts with the modulation of cell surface MICA in HSV1-infected cells, which is mediated by one or more late gene products of HSV1 [22]. Future research will need to identify which viral gene(s)/factor(s) are responsible for the detected ligand downregulation in PRV-infected cells. Although the exact mechanisms of PRV-induced NKG2D ligand downregulation remain to be investigated, our current data indicate that this downregulation appears to be based on at least two modes of action. On the one hand, PRV infection prevents mRNA production and cell surface protein expression of new NKG2D ligands, while at the same time triggering the downregulation of ligands already present on the cell surface. It will be interesting to investigate in future assays whether suppression of NKG2D ligand mRNA expression can be explained by the general host gene expression shutdown reported for herpesviruses [21,52,53], whether newly produced NKG2D ligand proteins may be retained in particular intracellular organelles and/or whether downregulation of pre-existing ligands may be due to activation of particular internalization processes [20]. The finding that PRV uses at least two different mechanisms to interfere with NKG2D ligand expression on the surface of infected cells suggests that NKG2D evasion may be of particular importance for the virus. Future experiments, including the generation of mutant PRV strains unable to trigger NKG2D ligand downregulation, will allow us to assess the importance of NKG2D evasion for PRV infection and spread in pigs.

Our results show that PRV infection results in a downregulation of pULBP1, the only confirmed porcine NKG2D ligand, as well as of pMIC2, a potential additional porcine ligand [16,17]. The inhibition of pULBP1 mRNA expression in PRV-infected cells may contribute to the observed reduced cell surface expression of pULBP1, as lower mRNA levels typically translate into lower ligand cell surface expression. However, the expression of mRNA for NKG2D ligands may not always correlate well with the presence of the corresponding proteins on the cell surface level [12]. However, downregulation of pULBP1 on the surface of PRV-infected cells was very limited and is therefore unlikely to explain the very strong downregulation of NKG2D binding. Therefore, we hypothesize that additional, possibly undiscovered NKG2D ligands are expressed on the surface of porcine cells, which would be in line with the multiple NKG2D ligands described in human and mouse cells.

Since our studies made use of human recombinant NKG2D for binding assays, the current findings may also be of relevance in the field of xenotransplantation. Xenograft rejections remain a major problem in xenotransplantation, due to interspecies immunological incompatibilities, which pose a barrier, and in which host NK cell-mediated graft rejections is of particular concern [54,55,56,57]. NKG2D and NKp44 have both been reported as important activating receptors involved in the activation of human NK cells by porcine cells [58,59]. Identifying the viral factors responsible for PRV-induced downregulation of porcine ligands for human NKG2D may therefore be relevant in future strategies to downregulate these ligands in porcine tissues considered for xenotransplantation. In line with such strategies, it has been reported that the expression of HCMV UL18, a viral analog of MHC class I that is able to transmit inhibitory signals to NK cells, in porcine endothelial cells suppressed the activity of human NK cells and prevented the killing of transduced porcine cells [60].

Our current data add new insights to our understanding of the interaction between alphaherpesviruses, particularly PRV, and NK cells and support the notion that many herpesviruses interfere with the recognition of infected cells by the activating NK cell receptor NKG2D, thereby suggesting that the NKG2D signaling axis may have put a substantial selective evolutionary pressure on these pathogens.

## Figures and Tables

**Table 1:** Primary and secondary antibodies used for cell surface expression analysis by flow cytometry.

Antigen	Clone	Isotype/Recombinant	Labeling Strategy
PRV gB [29]	1C11	IgG_2a_	Secondary antibody R-phycoerythrin (R-PE) conjugated goat anti-mouse IgG (Invitrogen, catalog number P-852)
PRV gD [29]	13D12	IgG_1_	Secondary antibody R-PE conjugated goat anti-mouse IgG
NKG2D ligands		Recombinant human NKG2D Fc Chimera protein (R&D, catalog number 1299-NK-050)	Secondary antibody R-PE-conjugated Goat Fab anti-human IgG (Invitrogen, catalog number H10104)
pULPBP1 [17]		IgM	Secondary antibody Alexa fluor 647 goat anti-mouse IgM (Invitrogen, catalog number A-21238)

**Table 2:** Forward and reverse primers used for RT-qPCR analyses.

Gene ^1^ (Encoded Protein, GenBank Accession No.) (Reference)	Sequence (5′-3′)	
	Forward	Reverse
pULBP1 (ULBP1, NM_001004035.1)	5′-TGG CTT GTG GAA GAT CCG AC-3′	5′-CCT GAG GTT CTG TGA TGG CA-3′
pMIC2 (MIC2, XM_003128306.4)	5′-AAC GGC TTC ACA CCA GAG AC-3′	5′-TTG AAT TCA GGC CTC CTG GC-3′
28S rRNA [30]	5′-GGG CCG AAA CGA TCT CAA CC-3′	5′-GCC GGG CTT ACC CAT T-3′

^1^ All genes are for the pig (*Sus scrofa*).

## Data Availability

The data presented in this study are available in Supplementary Materials.

## Supplementary Materials

The following are available online at https://www.mdpi.com/1999-4915/13/2/266/s1, Figure S1: NKG2D binding assay with an addition Fc-only recombinant protein control. Figure S2: UV-inactivated PRV is unable to trigger downregulation of NKG2D ligands.
