# Supplementary material for: Pseudorabies Virus Infection Causes Downregulation of Ligands for the Activating NK Cell Receptor NKG2D
Source: Viruses. 2021 Feb 9;13(2):266. doi: 10.3390/v13020266 (PMC7915010; doi:10.3390/v13020266)
Supplement: Supplementary file 1 [file viruses-13-00266-s001.pdf]

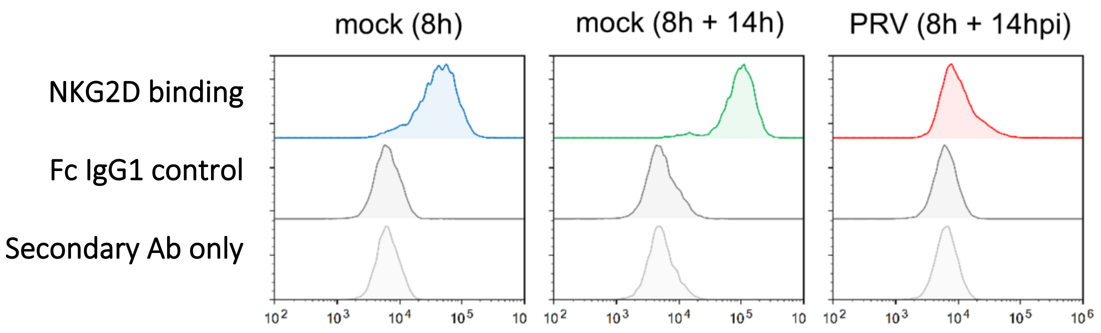

Figure S1. NKG2D binding assay with an addition Fc only recombinant protein control. SK cells were cultivated in suspension for 8 hours before mock or PRV inoculation. Immediately after the 8h cultivation (mock (8h)), or after an additional 14h upon mock inoculation (mock (8h + 14h)) or at 14hpi with PRV (PRV (8h + 14hpi)), cells were incubated with recombinant Fc-tagged human NKG2D protein (upper row), a secondary antibody control (middle row) or an Fc IgG1 control protein (lower row) and analysed by flow cytometry. Graphs show the median fluorescence intensity (MFI) for each condition.

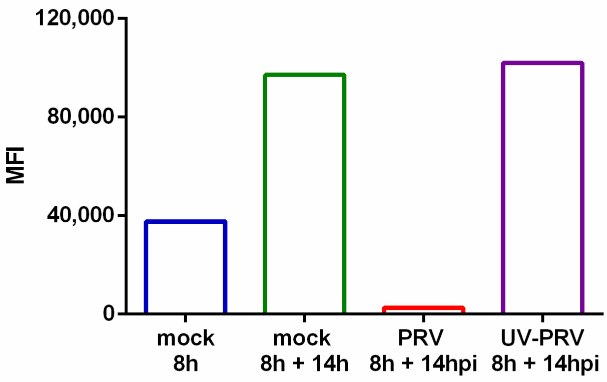

Figure S2. UV-inactivated PRV is unable to trigger downregulation of NKG2D ligands. SK cells were cultivated in suspension for 8 hours before mock, PRV or UV-inactivated PRV inoculation. Immediately after the 8h cultivation ('mock 8h'), or after an additional 14h upon mock inoculation ('mock 8h + 14h' or at 14hpi with PRV ('PRV 8h + 14hpi') or UV-inactivated PRV ('UV-PRV 8h + 14hpi'), cells were incubated with recombinant Fc-tagged human NKG2D protein and analysed by flow cytometry. Graphs show the median fluorescence intensity (MFI) for each condition.
